# Anti-homer-3 Antibody Encephalitis in a 10-Year-Old Child: Case Report and Review of the Literature

**DOI:** 10.3389/fneur.2022.929778

**Published:** 2022-06-13

**Authors:** Zuying Kuang, José Fidel Baizabal-Carvallo, Mohammad Mofatteh, Sifen Xie, Zhanhang Wang, Yimin Chen

**Affiliations:** ^1^Department of Neurology, Guangdong 999 Brain Hospital, Guangzhou, China; ^2^Parkinson's Disease Center and Movement Disorders Clinic, Department of Neurology, Baylor College of Medicine, Houston, TX, United States; ^3^Department of Sciences and Engineering, University of Guanajuato, León, Mexico; ^4^School of Medicine, Dentistry and Biomedical Sciences, Queen's University Belfast, Belfast, United Kingdom; ^5^Department of Neurology, Foshan Sanshui District People's Hospital, Foshan, China

**Keywords:** anti-homer-3 antibody, autoimmune encephalitis, antibodies, cerebellar ataxia, children

## Abstract

**Objective:**

We present a rare case with anti-Homer-3 antibodies positive encephalitis in the youngest patient ever identified and reviewed the literature.

**Case Report:**

A 10-year-old, Chinese boy came for evaluation of a 2-week history of cognitive impairment, irritability, dysarthria, and cautious gait. The neurological examination was consistent with the pan-cerebellar syndrome and encephalopathy. Cerebrospinal fluid (CSF) was inflammatory with increased leukocytes. Magnetic resonance imaging of the brain showed hyperintensities in both cerebellar hemispheres and vermis in Fluid-attenuated inversion recovery (FLAIR) and T2- weighted sequences. Infectious disorders were ruled out, but positivity for anti-Homer-3 antibodies was detected in the CSF, but not in the serum. Additionally, low titers of voltage-gated calcium channel (VGCC) antibodies were found in the serum. Treatment with intravenous (IV) corticosteroids did not provide meaningful clinical improvement; however, the patient achieved almost complete recovery (modified Ranking Scale score: 1) following IV immunoglobulin.

**Conclusion:**

Anti-Homer-3 cerebellar ataxia with encephalopathy should be considered within the differential diagnosis of acute inflammatory cerebellar disease in children and it may coexist with VGCC antibodies.

## Introduction

Cerebellar ataxia may be associated with genetic and acquired causes. Among the latter, an autoimmune or paraneoplastic etiology should be considered within the differential diagnosis. Anti-Homer-3 antibody encephalitis is a rare cause of autoimmune cerebellar ataxia first described in 2007 by Zuliani and colleagues, in a patient with subacute cerebellar ataxia (1). Anti-Homer-3 cerebellar ataxia is mostly described in adult patients, with the youngest patient being 14 years old (2). In this manuscript, we report a case of anti-Homer-3 cerebellar ataxia in a 10-year-old child, analyze the clinical profile of anti-Homer-3 autoimmunity, and contrast it with a closely related molecular disorder involving metabotropic glutamate receptor 1 (mGlutR1) autoimmunity.

## Case Report

A 10-year-old, right-handed, Chinese patient presented for clinical evaluation of a 2-week history characterized by impaired concentration, irritable mood, slurred speech, and slow, cautious gait. There was no recent history of an infectious disorder. The neurologic examination revealed bradypsychia, dysarthria, and downbeat nystagmus. The patient had dysmetria and dysdiadochokinesia. Tandem gait showed frank ataxia. Romberg's sign was negative.

**Figure 1:**
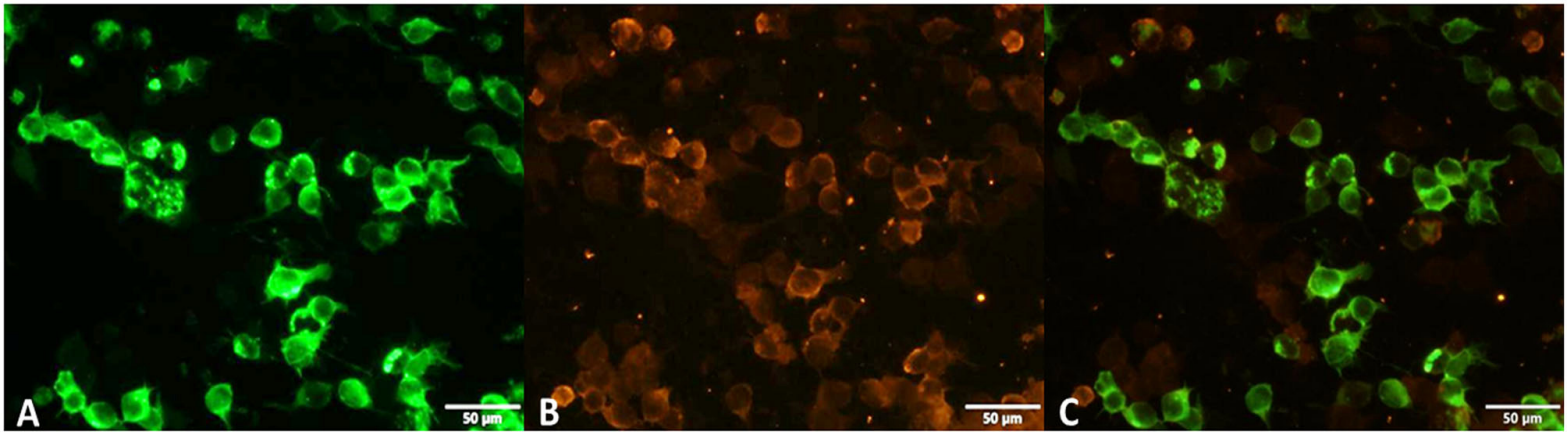
The anti-Homer-3 antibody of CSF was positive. **(A)** The green marker represents Homer-3 antigen, **(B)** the red marker represents anti-Homer-3 antibody; **(C)** the third one is fluorescence overlap. The images were taken using Olympus IX73 microscopy. The scale bar is 50 μm.

Initial investigation showed normal blood cell count, lactic acid levels, d-dimer assays, and hepatic and renal function tests. Serology for hepatitis C and HIV type 1 and 2 were negative. A lumbar puncture revealed an opening pressure of 16.5 cm/H_2_O. Cerebrospinal fluid (CSF) protein was 0.3 g/L (0.15–0.45 g/L), lactic dehydrogenase was 40.5 U/L ( ≤ 70 U/L), granular leukocytes were 30 × 10^6^/L (0–5 × 10^6^/L), and glucose and chloride levels were normal. Ziehl-Neelsen staining for acid-fast bacilli and Cryptococcus neoformans smear test in the CSF were negative. We used tissue-based assay by indirect immunofluorescence in fixed rat brain slices to screen for antibodies reacting with central nervous system antigens. Then, we used a commercially available cell-based assay to check for interest antibodies. Anti-Homer-3 antibody was positive in the CSF (1:1) (Figure 1) cell-based assay; however, the test was negative in the serum. Other antibodies such as anti-NMDAR, anti-AMPA1, anti-AMPA2, anti-CASPR2, anti-DPPX, anti-DRD2, anti-GlyR, anti-GAD65, anti-GABA_B_, anti-IgLON5, anti-LGI1, anti-mGlutR1, and anti-mGlutR5, as well as onconeural antibodies, were negative; however, low titers of anti-voltage gated calcium channel antibodies (VGCC-antibodies) were found in the serum: 41 pmol/L (normal value ≤ 30 pmol/L).

A detailed ultrasound examination of abdominal and pelvic structures was normal without evidence of tumor and a chest X-ray was unremarkable.

**Figure 2:**
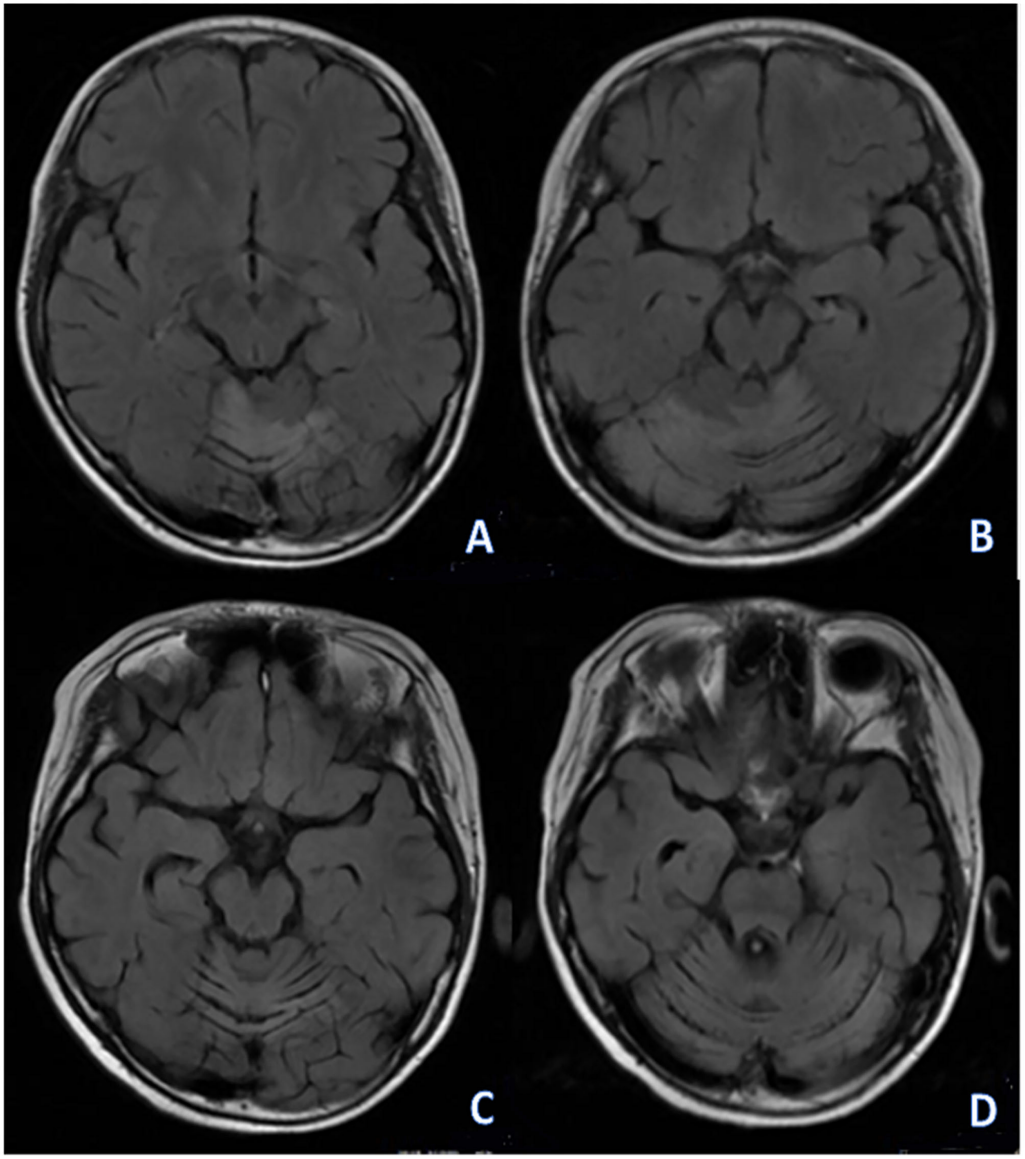
**(A,B)** initial MRI: right cerebellar atrophy and multiple lesions in bilateral cerebellum and vermis. **(C,D)** follow-up MRI showed bilateral cerebellum and vermis atrophy, sulcus of left cerebellum was wider.

Brain magnetic resonance imaging (MRI) showed hyperintensities in both cerebellar hemispheres and vermis in FLAIR and T2-weighted (T2W) sequences (Figures 2A,B).

Initial treatment was carried out with intravenous boluses of methylprednisolone (1 g per day for 5 days), followed by oral prednisolone at decreasing doses for 15 days. No improvement was observed on day 10; for that reason, intravenous immunoglobulin (IVIg) was offered, but the patient's parents initially declined for personal reasons.

As no improvement in cognitive and motor condition was observed in the following weeks, the patient's parents accepted treatment with IVIg 1 month after the initial assessment. Slow but progressive clinical improvement was noticed with normalization of cognitive impairment and cerebellar syndrome a year later March 1, 2022, when only mild dysarthria was noticed, a score of 1 on the modified Rankin Scale was registered at that time. Follow-up MRI showed mild cerebellar atrophy (Figures 2C,D).

## Discussion

We report the youngest case ever with autoimmune encephalitis associated with anti-Homer-3 antibodies in a 10-year-old child, manifested by encephalopathy, cerebellar ataxia, and hyperintensities in the cerebellum. Acute and subacute cerebellar ataxia may follow diverse etiologies in children, including nutritional, toxic, post-infectious (e.g., Epstein-Barr, herpes simplex, measles, mumps, parvovirus B19, varicella, Mycoplasma, etc.), or autoimmune. Most of these causes do not show signs of inflammation in the cerebellum when assessed by MRI (3). However, few cases of post-infectious cerebellitis may present with cerebellar hyperintensities on MRI followed by cerebellar atrophy (4). Patients with post-infectious cerebellar ataxia usually show a prodromal period with a febrile illness, followed by frank cerebellar ataxia; this was not observed in our patient. Moreover, acute cerebellitis in children may be observed with metronidazole and vigabatrin therapy, these patients show a distinctive pattern on MRI with pons and dentate nuclei hyperintensities (5). While a paraneoplastic or an ischemic etiology is more commonly seen in adults with acute cerebellar ataxia, these etiologies are less common in children, which varies the clinical approach. Autoimmune cerebellar ataxia in children may be related to anti-glutamic acid decarboxylase (anti-GAD) antibodies, gluten ataxia, or the cerebellar variant of Hashimoto's encephalitis, but other etiologies have been rarely described (3).

Most patients reported with anti-Homer-3 autoimmunity are adults with cerebellar ataxia as the most common presentation; however, other neurologic manifestations, such as encephalopathy, seizures, myeloradiculopathy, radiculoneuropathy, autonomic neuropathy, and REM-sleep behavior disorder (RBD), have been described (Table 1) (6–8). Although the number of reported cases in the literature is small, multiple system atrophy cerebellar subtype C (MSA-C)-like syndrome with brainstem and cerebellar atrophy along with RBD has been reported in adults; whereas inflammation with diffuse hyperintensities in the cerebellum has been only observed in young individuals: 14 and 10-year-old patients as described in this report (Table 1).

**Table 1:** Summary of reported clinical cases of anti-Homer-3 associated autoimmunity.

**References**	**Sex/age (years)**	**Onset**	**Main manifestations**	**MRI findings**	**Treatment**	**Outcome**
Zuliani et al. (1)	F/65	Subacute	CA, vertigo, vomiting	Normal	Steroids	No benefit
Höftberger et al. (6)	M/38	Acute	CA, encephalitis, seizures, papilledema vomiting	Cerebellar atrophy (f-u)	Steroids, IVIg	Partial recovery
Xu et al. (7)	F/51	Insidious	CA, dizziness, RBD	Cerebellar atrophy (f-u) Hot cross Bun sign	Steroids, MMF	Partial recovery
Liu et al. (8)	F/46	Insidious	CA	Cerebellar atrophy (f-u)	Steroids, MMF	Partial recovery
	M/14	Subacute	CA, encephalitis, myeloradiculopathy	Diffuse cerebellar T2W hyperintensities	Steroids, IVIg	Partial recovery with relapses
	M/65	Insidious	CA, RBD	Cerebellar and pons atrophy (f-u) Hot cross Bun sign	Steroids, IVIg, PE	No benefit with deterioration
	F/84	Subacute	CA	Normal	Steroids	Stability
	F/69	Subacute	CA, encephalopathy, radiculoneuropathy	Diffuse cerebral (FLAIR) hyperintensities	IVIg, steroids	Partial recovery with relapses
This report	M/10	Acute	CA, encephalopathy	Diffuse cerebellar T2W hyperintensities; cerebellar atrophy (f-u)	Steroids, IVIg	Almost complete recovery

*CA, cerebellar ataxia; F-u, follow-up; MMF, mycophenolate mofetil; RBD, REM-sleep behavior disorder*.

The Homer-3 antigen is enriched in the dendritic spines of cerebellar Purkinje cells and is also expressed in their cell bodies and axons (9). The cerebellar cortex, hippocampus, and non-neuronal tissues (e.g., thymus and lung) also express the Homer-3 antigen (9). Homer-3 interacts with the metabotropic glutamate receptor 1 (mGluR1) C-terminus in Purkinje cells to regulate its trafficking and clustering in the cell membrane to modulate its functional activity (9). Autoimmunity related to anti-mGluR1 antibodies has been described in a larger number of patients; there are several similar features between anti-Homer-3 and anti-mGlutR1 autoimmunity (Table 2), suggesting biological convergence between both disorders. However, it should be noticed, that while there is compelling *in vitro* and *in vivo* evidence that anti-mGlutR1 autoimmunity is related to pathogenic antibodies (1, 10), anti-Homer-3 autoimmunity is more likely to be related to cellular toxicity as the antigen is intracellular.

**Table 2:** Differences between Homer-3 and mGluR1 autoimmunity.

	**Homer-3**	**mGluR1**
Age range (years)	10–84	6–81
Median age at onset (years)	51	55
Sex distribution (female)	55.5%	43%
Paraneoplastic association	0%	11%
Underlying neoplasm	None reported	Hodgkin lymphoma Cutaneous T-cell lymphoma
Main manifestations	CA isolated or combined with encephalopathy, RBD (MSA-like), myeloradiculoneuropathy	CA isolated, behavior/cognitive changes, dysgeusia. dysautonomia, MDS*
Imaging patterns on MRI	Cerebellar atrophy Cerebellar and brainstem atrophy Cerebellar hyperintensities	Normal Mild cerebellar atrophy Cerebellar hyperintensities
Cerebellar hyperintensities or meningeal enhancement	22.2%	16%
Treatment	Steroids, IVIg, PE, MMF	Steroids, IVIg, PE, Aza, RTx, HQQ
Outcome	Partial recovery sometimes with relapses. No improvement in some instances	Most patients have marked improvement

*MDS: chorea, dystonia, tremor. Aza, azathioprine; CA, cerebellar ataxia; HQQ, hydroxychloroquine; IVIg, intravenous immunoglobulin; MMF, mycophenolate mofetil; PE, plasma exchange; RBD, REM-sleep behavior disorder; RTx, Rituximab.

Patients with anti-Homer-3 autoimmunity seem to have a worse prognosis and lower response rate to immunotherapy than patients with anti-mGlutR1 antibodies (Table 2), although direct comparisons are lacking (10, 11). A previous study reported that among six patients with anti-Homer-3 autoimmunity who received immunotherapy, four improved, but still showed residual disability with a modified Ranking Scale ≤ 2 at the last follow-up; moreover, relapses, might present following initial remission or improvement with immunosuppressant (8). However, whether all patients with anti-Homer-3 autoimmunity should undergo prolonged immunosuppression with medications, such as azathioprine or mycophenolate mofetil, is unclear.

Voltage-gated calcium channel antibodies are associated with Lambert-Eaton myasthenic syndrome, characterized by proximal muscle weakness, areflexia, and autonomic dysfunction; none of these features were observed in our patient (12). Anti-VGCC antibodies are paraneoplastic in about 90% of cases in the context of cerebellar ataxia, mainly associated with small cell lung cancer (SCLC); there was no evidence of underlying neoplasm in our patient. Some patients with positive anti-VGCC antibodies may present with non-paraneoplastic cerebellar degeneration, responding to immunotherapy (13). We cannot rule out that these antibodies contributed to the clinical manifestations in our patient; however, much higher serum titers between 500 and 648 pM/L have been reported in patients with paraneoplastic and non-paraneoplastic cerebellar ataxia (13, 14); moreover, we are not aware of VGCC autoimmunity presenting with signs of cerebellar inflammation in the MRI.

## Conclusions

Autoimmunity related to anti-Homer-3 is mostly identified in adults, but may also present in children. Although data is scarce, it seems that in this age group, patients present with signs of cerebellar inflammation on MRI preceding cerebellar atrophy, contrasting with cerebellar, and brainstem atrophy observed at presentation and during the disease in adult patients. Anti-Homer-3 autoimmunity should be considered within the differential diagnosis of autoimmune cerebellar ataxia in childhood. Negative serum antibodies do not rule out the disorder as anti-Homer-3 antibodies may be present only in the CSF.

## Data Availability Statement

The raw data supporting the conclusions of this article will be made available by the authors, without undue reservation.

## Ethics Statement

Ethical review and approval was not required for the study on human participants in accordance with the local legislation and institutional requirements. Written informed consent to participate in this study was provided by the participants' legal guardian/next of kin.

## Author Contributions

YC, ZK, ZW, and SX conceived the study, gathered the data, and drafted the manuscript. JB-C and MM conceived the study, review, and critique. All authors contributed to the article and approved the submitted version.

## Conflict of Interest

The authors declare that the research was conducted in the absence of any commercial or financial relationships that could be construed as a potential conflictof interest.

## Publisher's Note

All claims expressed in this article are solely those of the authors and do not necessarily represent those of their affiliated organizations, or those of the publisher, the editors and the reviewers. Any product that may be evaluated in this article, or claim that may be made by its manufacturer, is not guaranteed or endorsed by the publisher.
